# Does Hippocampal Volume Predict Transition to Psychosis in a High-Risk Group? A Meta-Analysis

**DOI:** 10.3389/fpsyt.2020.614659

**Published:** 2021-01-14

**Authors:** Bernd Hinney, Anna Walter, Soheila Aghlmandi, Christina Andreou, Stefan Borgwardt

**Affiliations:** ^1^Department of Psychiatry (UPK), University of Basel, Basel, Switzerland; ^2^Basel Institute for Clinical Epidemiology and Biostatistics, University Hospital Basel, Basel, Switzerland

**Keywords:** schizophrenia, psychosis, hippocampus, neuroimaging, high risk, at-risk mental state

## Abstract

Schizophrenia has a prodromal phase of several years in most patients, making it possible to identify patients at clinical high risk (CHR) for developing the disorder. So far, these individuals are identified based on clinical criteria alone, and there is no reliable biomarker for predicting the transition to psychosis. It is well-established that reductions in brain volume, especially in the hippocampus, are associated with schizophrenia. Therefore, hippocampal volume may serve as a biomarker for psychosis. Several studies have already investigated hippocampal volume in CHR groups. Based on these studies, the present meta-analysis compares the baseline left and right hippocampal volume of CHR patients who developed a psychosis with that of CHR patients without such a transition. Our results show no statistically significant effect of the hippocampal volume on the transition risk for psychosis.

## Introduction

Schizophrenia is a psychiatric illness that is typically preceded by a long prodromal phase (1). The disease has a significant impact on patients' quality-adjusted life years and an often unsatisfactory response to treatment (2). One of the main goals of psychiatric public health government programs is to establish criteria for early detection of schizophrenia. Such criteria could make it possible to implement various forms of secondary prevention to avoid or delay the onset of schizophrenia (3). A large number of studies have accordingly followed-up clinical high-risk (CHR) groups in order to investigate the factors associated with later development of a psychotic disorder (among them the NAPLS- and the PRONIA-project) (4–8). CHR-status is identified according to specific clinical criteria; the most widely established criteria define high-risk based on either (9–12).

a) “attenuated psychotic symptoms” (ASP) or “brief limited intermittent psychotic symptoms” (BLIPS) (these symptoms are manifestations which are typical for psychotic disorders, but which are either not sufficiently severe or too short to warrant the diagnosis itself)
b) genetic vulnerability for psychotic disorders accompanied by a notable downward shift in an individual's social functioning (measured, for example, by difficulties at work or by the inability to live autonomously).

In practice, criterion b is of little relevance in the actual setup of a concrete CHR-group (13). Thus, the dominance of clinical features further highlights the status of the CHR-group as a representation of the prodromal phase.

When CHR patients develop a psychotic disorder, this is called a “transition.” The probability of transition was initially estimated to be between 35 and 40% in typical CHR-groups (14), but in recent studies, it was lower (15). This could be because of specific protective effects that arise from a diagnosis of CHR: for example, increased risk-awareness of the included person and support from family members, school officials, or other professionals (16).

The low transition rates in CHR-groups mean that it is not feasible to undertake a prophylactic treatment in all CHR patients, especially if such a treatment encompasses the use of antipsychotic medication with the accompanying side-effects.

To enhance the predictive value of CHR-criteria, it has been hypothesized that the inclusion of neuro-imaging data should be helpful (3, 17–19). Schizophrenia is known to be accompanied by volume enlargements in the ventricular regions (especially in the later stages of the disease) (20) and volume reductions in several brain areas, among them the frontal cortex, the amygdala, the parahippocampal gyrus and the hippocampus (21–37). It has also been suggested that these changes are progressive during the course of the illness (38). Especially hippocampal volume reductions might not only be a result of the disease, but instead have been suggested to be one of its causative factors (39–42). Such causative relationships might be explained by the immense role of the hippocampus in memory formation (43, 44). Hippocampal volume reductions and ensuing memory deficits could lead to false hypothesis-testing in CHR-individuals (caused for example by a functional shift from pattern separation to pattern completion) and this might make the individual more prone to psychosis (45). Should such neuroanatomical processes occur, lower hippocampal volumes (compared to healthy controls) should be observable before the actual onset of schizophrenia, which means during the prodromal phase. Such volumetric changes could be a useful biomarker and supplement the predictive value of risk-assessments based on the clinical criteria mentioned above, thus increasing the specificity of the CHR concept (46).

Several studies have carried out MRI-measurements of the hippocampus on members of CHR-groups, and have then compared the results to a parallel arm of healthy controls (47–57). The goal of these studies was to assess whether CHR patients have smaller hippocampal volumes compared to healthy controls *at baseline* (i.e., at the time of diagnosis of the CHR status) compared to healthy individuals. A selection of those studies has been pooled in a meta-analysis by Walter et al., which did not find any significant differences between CHR patients and controls (58). Following up on this finding, the aim of the present meta-analysis is to compare CHR-patients who made the transition to psychosis during the follow-up interval (“converters”) with CHR-patients who did not (“non-converters”). This concept has first been employed in a study by Pantelis et al. (59). In the context of our meta-analysis, this procedure implies ignoring the control group of the investigated studies and retrospectively splitting up the case group (CHR patients) into two groups, defined by their transition status. Cases and controls are thus generated from the same cohort, e.g., the CHR-population. Methodologically, this has the significant benefit of reducing heterogeneity between the groups, which is a frequent challenge in case-control studies. Also, from a clinical viewpoint, it is a meaningful approach to remove a potential “dilution” effect that may have occurred in previous studies when combining converters and non-converters in one group.

## Materials and Methods

### Eligibility Criteria

We included studies in our meta-analysis if they investigated individuals that met CHR-criteria. Studies had to assess CHR-status according to established criteria and the minimum follow-up period was to be 12 months, as adequate transition rates can be expected after this time (14). Another important factor for eligibility was the presence of the hippocampal volume as an absolute value and not in relation to other parts of the brain [as in voxel-based measurements (VBM)] because the latter values are not ideal for direct statistical comparison and may be compromised through position changes during image registration (19, 60).

We assembled eligibility criteria according to the PRISMA-P guidelines. The following paragraph summarizes the inclusion and exclusion criteria that we applied:

Inclusion criteria:

1. Risk status established according to international research diagnostic criteria for high clinical risk for psychosis (CAARMS, BSIP, PACE, SIPS/SOPS) or primary symptoms (SPIA)
2. Hippocampal volume obtained through the region of interest (ROI) analysis (manually tracing or automated segmentation) reported separately for members of the transition-group and the non-transition group
3. Availability of mean values (±SD) of left and right hippocampal volume
4. Publication in a peer-reviewed journal
5. Follow-up interval of at least 12 months.

Exclusion criteria:

1. A sample size of <10 participants
2. Comorbidity with medical or neurological illnesses in patients
3. Any post-mortem assessments
4. Studies on the chromosome 22q11.2 deletion syndrome.

We registered the study with PROSPERO in February 2019, and our study project was approved in April 2019.

### Search Strategy and Selection Process

We conducted a systematic literature search in the public databases Medline and EMBASE on April 28, 2019. We repeated the search on May 28, 2019. We used the following search terms: “(MRI OR magnetic resonance imaging OR neuroimaging) AND (psychos^*^ OR schizophrenia^*^ AND high-risk OR at-risk mental state OR prodrom^*^) AND hippocamp^*^”.

We removed duplicates using EndNote and two authors (BH and AW) screened the references based on the titles and abstracts. All potentially relevant references were read in full-text and independently assessed by two authors (BH and AW). We resolved any disagreements by consensus. To identify potential additional studies that were not included in electronic databases, we screened the bibliographic references of all included articles.

In this fashion, we recovered 157 papers. Two additional articles were found by screening the references. One hundred and fifty six papers remained after removing duplicates. One hundred and forty six papers could be removed because they were not relevant to our topic or included reprints. Of the 10 remaining articles, we excluded one because it recorded hippocampal volume as a VBM-measurement (61) and we excluded one other study because its sample overlapped with a newer study that better fitted our inclusion criteria (57). In three additional cases, authors did not reply to our request for complete data extraction (47, 54, 56).

**Figure 1:**
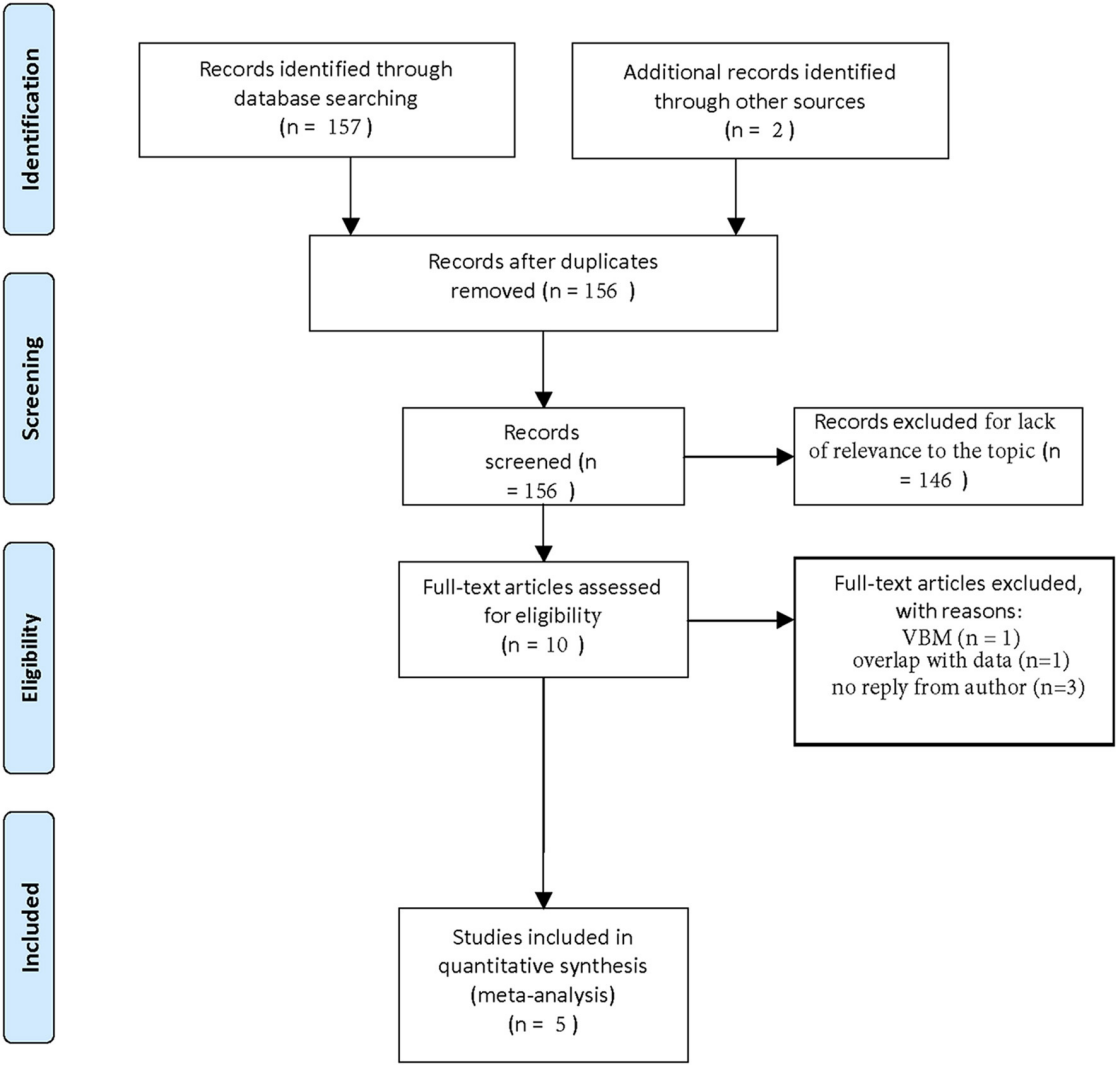
Flow chart of selection process.

In this process, we could filter out five studies that could be included in our meta-analysis (48, 50, 52, 53, 55). The selection process is visualized in a flow-chart (see Figure 1).

### Data Extraction

Necessary information was extracted by one reviewer (BH) and independently checked by a second reviewer (AW) and entered into a Numbers database. We identified and resolved discrepancies through discussion. Data items to be extracted were: first author name, the cohort, publication date, MRI resolution, hippocampal volume right/left (with standard deviation), number of men/women and mean age of participants (with standard deviation). In some cases, we contacted the authors of the original study in order to provide for the missing variables.

Hippocampal volume can be presented in two formats: as a “raw,” uncorrected number or corrected for the intracranial volume (ICV). There is no consensus in the scientific community which format is more adequate. Hippocampal volume correlates with ICV, but this correlation is not proportional (62). Our goal was to carry out the statistical analysis with both the corrected and the uncorrected values; however, in two cases we could only retrieve the uncorrected values from the authors. All hippocampal volumes stated in this paper are thus the raw volumes in mm^3^. They represent the mean values of the individual hippocampal measurements in each group (transition/non-transition-group).

### Data Synthesis

We carried out a meta-analysis to assess the differences in the sizes of the left and right hippocampus in a CHR-group for psychosis. Using the data extracted from each paper (see section above), we built an evidence table to investigate possible between-study heterogeneity.

We then used the random-effects model to account for potential statistical heterogeneity, reporting the standardized mean difference and the respective 95% confidence interval (CI). We used standardized mean differences (SMD) due to the diversity of MRI-devices used (e.g., different resolutions) and applied the Hedges' g method (63). For calculating the pooled SMD using a random-effect model, we used the Dersimonian-Laird estimator for estimating the between-study variance (τ^2^) (64). Forest plots were generated to show the individual and pooled effect measure, 95% confidence intervals (CI), the author's name, and study weights for the studies.

We assessed heterogeneity between the results of the studies by using the Cochran's Q test and quantified heterogeneity with the I-squared statistic. Heterogeneity based on the I-square is considered low, moderate, or high when the values are below 25%, between 25 and 75%, or above 75%, respectively.

We investigated sources of inter-study heterogeneity with the univariable random-effects meta-regression analysis that is based on the following primary study characteristics: year of publication, sample size, gender ratio, and MRI resolution. We weighted meta-regression analyses to account for both within-study variances of treatment effects and the residual inter-study heterogeneity.

We performed all analyses using the *metacont* function of the *meta* package in R 3.6.1 (R Foundation for Statistical Computing, Vienna, Austria) (65).

## Results

### Systematic Review

All five studies that we could include in our meta-analysis were designed as case-control studies in which a healthy control group was compared to a CHR-group for psychosis in terms of baseline volumetric MRI-measurements. After baseline, the CHR-group was clinically followed up to determine transition status. As described above, we ignored the control-group in our analyses and only focused on the CHR-group.

In the following systematic review, we summarize and analyze the five studies regarding the way they established data for CHR-groups and regarding further study characteristics. We do not report special inclusion or exclusion criteria that are already understood due to the requirements we set for our meta-analysis (see section “Materials and Methods”).

*Dean et al*. established CHR-status in an US-American sample using the “Structured Interview for Prodromal Syndromes” (SIPS) (48). Patients who had received antipsychotic medication before the interview were excluded, as were those with a lifelong drug dependence. Two members of the CHR-group who received antipsychotic medication between inclusion and follow-up (12 months later) were also excluded. This is noteworthy, as this may have underweighted the transition group. Reported drop-out rates in the CHR-group were low, with 16.7%. The study had a rather short follow-up period of 12 months, which can be assumed to have only captured a part of the actual scope of transition. Scanning was done on a 3-Tesla device and automated segmentation with FreeSurfer (version 5.3.0) was used for image processing.

*Cannon et al*. also used the SIPS for CHR-inclusion in a US-American sample. There were no special exclusion criteria for the use of antipsychotic medication or drug abuse, as the study was done in a naturalistic setting. Drop-out rates were not reported, and the follow-up periods were short with 12 months (52). Scanning was performed on a 3-Tesla device and automated segmentation with FreeSurfer (version 5.2.0) was used for image processing.

*Pruessner et al*. used the “Comprehensive Assessment of At Risk Mental States” (CAARMS)-interview to establish CHR-status in a Canadian sample. Anyone with a history of substance use disorder or mental illness was excluded. Drop-out rates were not reported, and the regular follow-up interval was 2 years (55). Scanning was performed on a 1,5-Tesla device and automated segmentation with a self-designed tool (66) was used for image processing.

*Harrisberger et al*. used the “Basel Screening Instrument for Psychoses” (BSIP) in a Swiss sample to establish CHR status (53). Only antipsychotic-naive individuals were included in the CHR group. Drop-out was not reported, and the follow-up interval was 3 years. Scanning was performed on a 3-Tesla device and automated segmentation with the software FSL-FIRST (67) was used for image processing.

*Buehlmann et al*. used the “Personal Assessment and Crisis Evaluation” (PACE)-criteria for CHR-status in a Swiss sample (50) (there was no overlap with the study by Harrisberger et al.). The study was naturalistic and did not require any specific inclusion or exclusion criteria beyond those generally required for inclusion in our meta-analysis. Drop-out rates were not reported, and the follow-up interval was 3 years. Scanning was performed on a 1,5-Tesla device and manual tracing combined with AMIRA was used for image processing.

### Participant Characteristics

The transition group (case group) consisted of 94 members (60 men, 34 women). The mean age was 21.57. The standard deviation of age (transition group) in the individual studies ranged from 2 to 7 years (see Table 1 for details).

**Table 1:** Evidence table.

			**Transition group**				**Non-transition group**			
**Author**	**Year**	**Cohort**	***N* men**	***N* women**	**Mean age**	**SD age**	***N* men**	***N* women**	**Mean age**	**SD age**
Dean	2016	Boulder	2	1	19.33	2.08	20	15	18.89	1.39
Pruessner	2017	Montreal	2	2	21.14	5.1	11	11	19.97	3.15
Buehlmann	2010	Basel	11	5	26.4	6.5	11	10	23.4	6
Cannon	2015	NAPLS	36	19	19.18	3.77	213	147	19.78	4.23
Harrisberger	2016	Basel and Zurich	9	7	25.56	7.31	50	25	23.22	4.35

The non-transition group (control group) consisted of 513 members (305 men, 208 women). The mean age was 20.38. The standard deviation of age in the individual studies ranged from 1 to 4 years (see Table 1 for details).

### Results of Meta-Analysis

Due to the observed heterogeneity between the studies, we only report the results for the random-effects meta-analysis.

**Figure 2:**
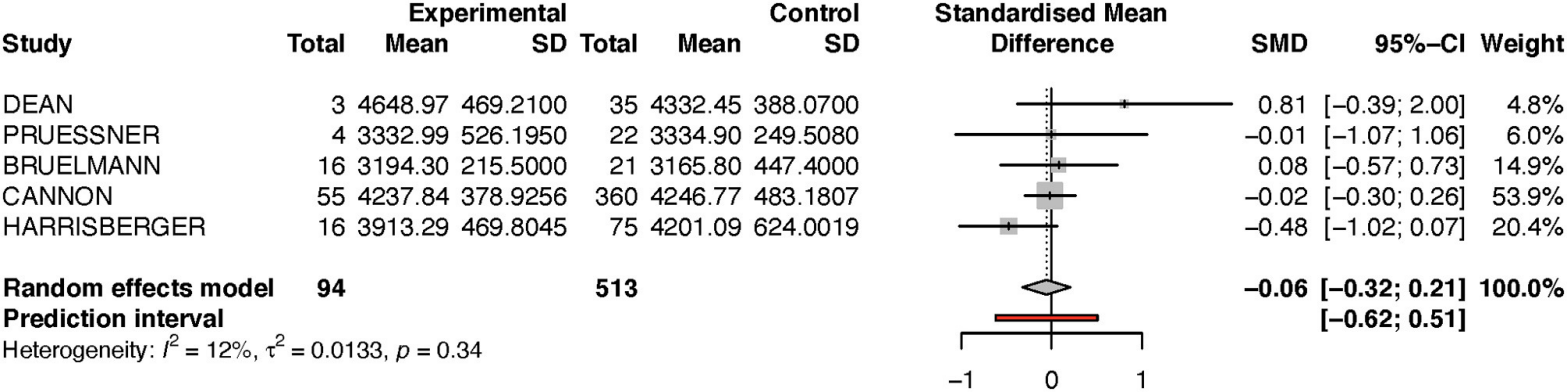
Forest plot for Right hippocampus (full analysis).

Looking at the right hippocampus, the standard mean difference shows that the volume of the right hippocampus is 6% less in the transition group than in the non-transition group (see Table 2A and Figure 2). The heterogeneity (*I*^2^) between the studies is 12%, which indicates low heterogeneity.

**Table 2A:** Summary of group-diffferences between transition-group and non-transition group for each study (right hippocampus).

**Author**	**Standardized mean difference**	**95% confidence interval**	**Weight (%)**
Dean	0.7885	[−0.4054; 1.9823]	4.5
Pruessner	−0.0062	[−1.0716; 1.0592]	5.7
Buehlmann	0.0761	[−0.5746; 0.7268]	14.4
Cannon	−0.0189	[−0.3027; 0.2648]	55.6
Harrisberger	−0.475	[−1.0193; 0.0693]	19.8

*“Weight” refers to the statistical weight within the random effects model*.

**Figure 3:**
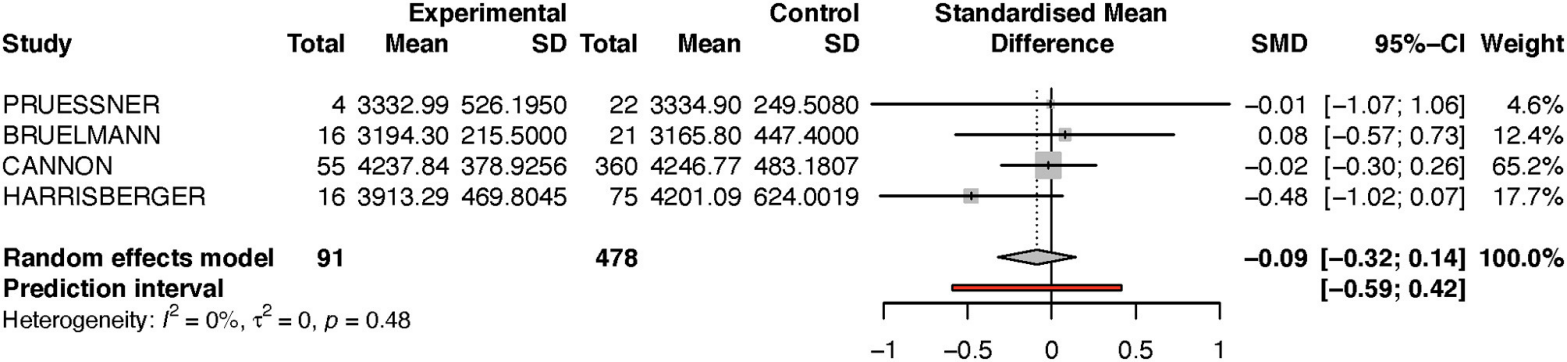
Forest plot for Right hippocampus (sensitivity analysis).

Because the study of Dean et al. had a very small sample size, and because its results diverged from that of the other studies, we performed a sensitivity analysis by excluding this one study (see Figure 3). Performing this analysis reduced heterogeneity to zero, and moved the *p*-value closer to the direction of rejection of the null-hypothesis. Nevertheless, in both analyses, the results were not statistically significant and indicate at most a numerical trend.

**Figure 4:**
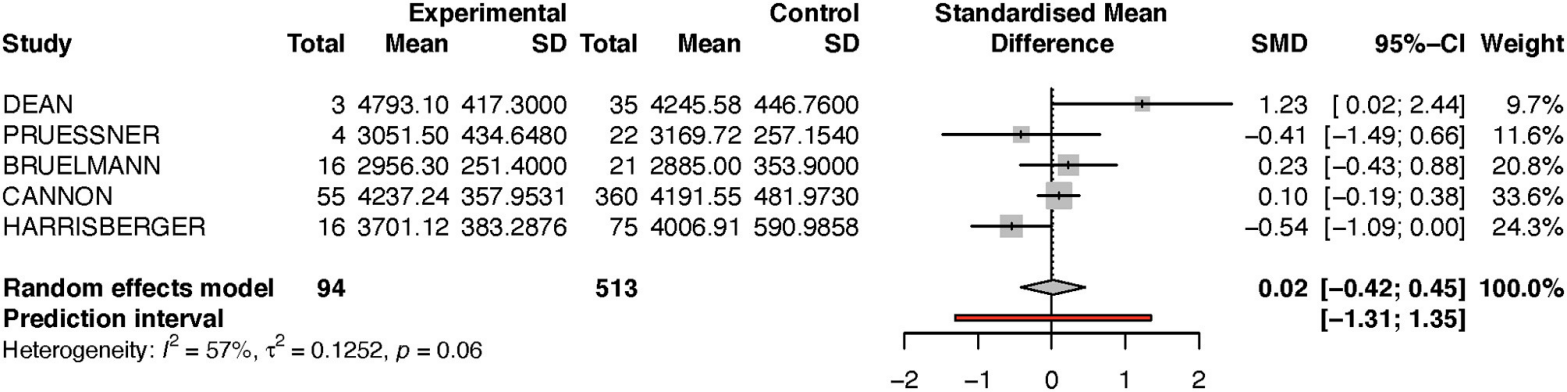
Forest plot for Left hippocampus (full analysis).

**Figure 5:**
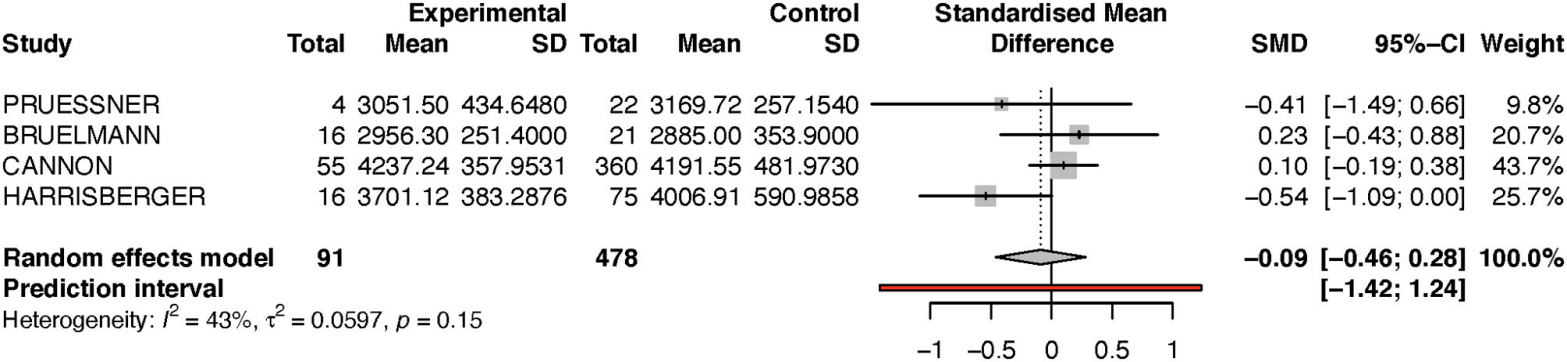
Forest plot for Left hippocampus (sensitivity analysis).

For the left hippocampus, the heterogeneity (*I*^2^) was much larger, with 57% (see Table 2B and Figure 4). The results have thus to be interpreted with caution, as is also indicated by the wide predictive interval. Initial analysis showed the left hippocampal volume to be 2% larger in the transition group. This is in contradiction to our hypothesis that the transition group has a smaller left hippocampus at baseline. However, when the sensitivity analysis was performed (again excluding the Dean et al. study, see Figure 5), this result was reversed, and the transition group's hippocampal volume was 9% smaller compared to the non-transition group. The results of both analyses for the left hippocampus are not statistically significant.

**Table 2B:** Summary of group-diffferences between transition-group and non-transition group for each study (left hippocampus).

**Author**	**Standardized mean difference**	**95% confidence interval**	**Weight (%)**
Dean	1.2041	[−0.0092; 2.4174]	9.5
Pruessner	−0.4011	[−1.4730; 0.6708]	11.4
Buehlmann	0.2221	[−0.4305; 0.8747]	20.7
Cannon	0.0975	[−0.1863; 0.3814]	34.1
Harrisberger	−0.5401	[−1.0858; 0.0056]	24.3

*“Weight” refers to the statistical weight within the random effects model*.

### Results of Meta-Regression

We performed a meta-regression for the following variables: year of publication, sample size, gender ratio and resolution of MRI-device (see Table 3). None of the assessed variables in the meta-regression significantly contributed to the between-study heterogeneity.

**Table 3:** Univariable meta-regression including all studies.

	**Right hippocampus**			**Left hippocampus**		
	**Estimate**	**95% CI**	***p*-value**	**Estimate**	**95% CI**	***p*-value**
Publication year (change per year)	−0.036	(−0.245, 0.174)	0.625	−0.065	(−0.382, 0.253)	0.562
Sample size (change per N)	−0.0001	(−0.004, 0.003)	0.943	−0.00001	(−0.006, 0.006)	0.982
Gender ration	0.138	(−1.620, 1.896)	0.818	0.194	(−2.613, 3.001)	0.84
MRI resolution	0.228	(−0.421, 0.878)	0.345	0.315	(−0.835, 1.464)	0.448

## Discussion

Our meta-analysis investigated whether hippocampal volume at baseline predicts the transition to psychosis in a CHR-group. Our study did not find a statistically significant relationship between these parameters. However, we did find a trend that points into such a direction when looking at the right hippocampus. This trend was further confirmed after performing a sensitivity analysis. The investigated studies showed much more heterogeneous results for the left hippocampus. Interestingly, a study by Seidman investigating the parahippocampal gyrus as a vulnerability factor for schizophrenia also found more pronounced effects for the right side of the brain (37).

It remains to be seen if future studies can corroborate this trend. If so, it will be interesting to see whether such a result is bilateral or whether the unilateral quality (e.g., the prominence of changes in the right hippocampus) still holds true.

Looking at the results of the systematic review, the most rigorous study-protocol was provided by two studies that excluded a history of mental illness and of drug abuse. Thus, the most important potential confounders regarding hippocampal volume were removed. Two other studies excluded either one or the other of those preconditions. Very few studies reported drop-out rates in the CHR-group, and follow-up intervals varied considerably. Transition rates are generally assumed to be 18% after a follow-up of 6 months, 22% after 1 year, 29% after 2 years, and 36% after 3 years (14). Thus, only two of the five studies we analyzed employed the upper limit regarding follow-up.

### Possible Clinical Implications

There is an ongoing debate about the concrete implications of risk predictions for members of CHR-groups. For a summary of the most important points in question, see Andreou and Borgwardt (19). For hippocampal volume to be of clinical use, a good cut-off margin between likely converters and non-converters will be required. This could provide clinicians with a biomarker that can be used to differentiate the CHR-group into an “extreme” risk group and a “reduced” risk group. Prophylactic treatment efforts could then be focused on those individuals who will most likely benefit from them.

### Limitations and Future Directions

Certain limitations of our meta-analysis need to be discussed. First of all, the overall number of studies was rather small. Second, looking only at the hippocampal areas may underestimate the complexity of the neuronal network that underlies the development of paranoid schizophrenia, and in addition McHugo recently noted that it may not be the whole hippocampus that changes in volume before and during psychosis, but rather specific subfields (especially the anterior hippocampus) (68). More precisely, it might be necessary to not only test left-right-asymmetry between global hippocampus volume (as was done in our study), but also to conduct a differential regional analysis (e.g., anterior or posterior region of the hippocampus) or a subfield analysis (e.g., subiculum, cornu ammonis, dentate gyrus).

Thirdly, follow-up periods varied between the studies, and short follow-up times may have led to some false-negatives within the non-transition-group. This would reduce the likelihood of discovering any significant relationship between (premorbid) hippocampal brain volume and the likelihood of developing psychosis. All of these factors could modify a relevant relationship between hippocampal volume and transition risk.

Methodologically, two of our studies scanned the brains of study participants on a 1.5 Tesla device while the other three used 3 Tesla devices. It is possible that the resulting differences in resolution affected the accuracy of the measurement of the hippocampus, although there generally seems to be a high correlation between 1.5 and 3 Tesla measurements of hippocampal volume (69, 70). Similarly, while four out of the five studies used differing versions of automated segmentation with FreeSurfer or other Software for the measurements of the hippocampal volume, the paper by Buehlmann et al. used manual segmentation. Regarding the image processing, the MRI-sequences used (T1 or T2) might also have an impact on the volumetric measurements, but this was a data point that was not available to us. It must also be noted that correction of raw hippocampal volume for ICV might have had a significant effect on the outcome.

Finally, none of the papers we analyzed explicitly mentioned the diagnostic instruments used to establish transition. While it can be assumed that transition to psychosis was usually confirmed by applying the ICD-10 and/or DSM-IV criteria for schizophrenia through an experienced clinician, it should be noted that ICD-10 and DMS-IV criteria for schizophrenia do differ slightly and that “transition to psychosis” is not in every case equivalent with the development of schizophrenia. Velakoulis et al. have noted that hippocampal volume loss may be much less pronounced in diagnoses of the schizophreniform spectrum than in schizophrenia itself (56). The diagnostic instruments used to establish CHR-status (CAARMS, BSIP, PACE, SIPS) also varied. Taken together, there are a number of potential sources of heterogeneity that should be considered when conducting further studies on the topic.

On a more general level, the concept of a macroscopically visible biomarker (e.g., shrinkages of the hippocampus in volumetric MRI-measurements) may not capture the reality of the prodromal phase. It is quite possible that during the prodromal phase, it is not the volume of a whole-brain structure that changes, but rather the way neuronal populations communicate with each other. Other techniques would have to be adopted to capture such changes, for example, MRT-assessments of functional connectivity as done by Blessing et al. (71). Also, volumetric assessments can be enhanced by measurements of cortical thickness, surface area, and gyrification to capture a wider range of potential changes in brains of individuals who go on to develop a psychosis (19). It is also possible to determine hippocampal volume at different time points (as was done by Cannon et al.) to determine a rate of change during the prodromal phase.

### Implications for Members of CHR-Groups Who Are Non-converters

An important question is whether CHR-members in the non-transition group can be regarded as healthy individuals or whether they constitute a subclinical set of the psychotic spectrum. While some of those individuals will eventually make the transition to psychosis (albeit after the follow-up interval), there will be a significant proportion of non-converters. For this group of individuals, essentially two etiological scenarios are possible:

a) non-converters carry the same risk for development of a psychosis as the members of the transition-group, but due to favorable circumstances (for example a very healthy life-style) psychosis does not manifest. This would be in accordance with the diathesis-stress-model of schizophrenia (72).

b) non-converters do not carry a relevantly heightened risk for the development of a psychosis. They are thus “wrongfully” included in the CHR-set due to the intentionally low specificity-levels of criteria used in the establishment of risk-groups.

Our study cannot answer the question which of these scenarios is most likely. It would be beneficial to include the subgroup mentioned in a long-term follow-up to gain more statistical information on their outcome and CHR-members should also be evaluated according to the healthiness of the lifestyle they adopt.

### Summary

In line with previous research, our meta-analysis indicates that there occur hippocampal volume changes in members of an ultra-high-risk-group before the transition to psychosis, but results did not reach significance thresholds. Larger samples are needed for future research in this area, and studies should not only look in more detail at the macroscopic level, but also assess the changes that occur on a functional level.

## Data Availability Statement

The original contributions presented in the study are included in the article/supplementary materials, further inquiries can be directed to the corresponding author/s.

## Author Contributions

SB and AW conceived the concept of the paper. BH and AW carried out the analysis of relevant literature. BH wrote the introduction, the results and the discussion section. BH and SA carried out the statistical analysis. SA wrote the Material and Methods section. CA revised the final manuscript. All authors contributed to the article and approved the submitted version.

## Conflict of Interest

The authors declare that the research was conducted in the absence of any commercial or financial relationships that could be construed as a potential conflict of interest.
